# Features of the Intestinal and Respiratory Microbiome in Colorectal Cancer Patients in Western Siberia

**DOI:** 10.3390/microorganisms14071392

**Published:** 2026-06-23

**Authors:** Vladimir G. Druzhinin, Elizaveta D. Baranova, Pavel S. Demenkov, Alexey S. Zhivotovskiy, Liudmila V. Matskova, Aleksey V. Larionov, Kirill S. Avdeev, Arseniy E. Yuzhalin

**Affiliations:** 1 Department of Genetics and Fundamental Medicine, Kemerovo State University, 650000 Kemerovo, Russiaalekseylarionov09@gmail.com (A.V.L.); avdeev.burzum@yandex.ru (K.S.A.); 2 Institute of Cytology and Genetics, Siberian Branch of the Russian Academy of Sciences, 630090 Novosibirsk, Russia; demps@bionet.nsc.ru; 3 M.S. Rappoport Kuzbass Clinical Oncology Dispensary, 650036 Kemerovo, Russia; 4 Research Institute of Molecular Biology and Biophysics, Siberian Branch of the Russian Academy of Sciences, 630117 Novosibirsk, Russia; 5 Research Center for Translational Medicine, Sirius University of Science and Technology, 354340 Sochi, Russia; 6 National Medical Research Center for Hematology, 125167 Moscow, Russia

**Keywords:** gut microbiome, respiratory microbiome, colorectal cancer, bacteria, 16S rRNA

## Abstract

To perform the first concurrent characterization of gut and respiratory microbiome profiles in colorectal cancer patients from Western Siberia, Russia. We analyzed synchronous fecal and sputum samples from 40 treatment-naive colorectal cancer patients and 45 healthy controls using 16S rRNA gene (V3-V4) sequencing and QIIME 2-based bioinformatic workflows. While alpha-diversity indices did not differ significantly between groups, beta-diversity analysis revealed substantial compositional differences for both ecosystems. Colorectal cancer patients exhibited gut enrichment of *Proteobacteria, Fusobacteria*, *Fusobacterium*, *Odoribacter*, *Lachnospiraceae_UCG*-010, *Erysipelatoclostridium*, *Parvimonas*, *Finegoldia*, *Clostridium* and *Bacteroides* (*Bacteroides fragilis*), alongside sputum enrichment of phyla Bacteroidetes and Actinobacteria, as well as genera *Neisseria*, *Prevotella*, *Lactobacillus*, *Rothia*, *Nocardia*, *Leptotrichia*, *Campylobacter*, and *Helicobacter*. Stage-associated shifts included elevated *Akkermansia* in gut microbiomes of patients with advanced-stage disease and higher *Campylobacter* in early-stage sputum. These findings identify distinct gut–respiratory dysbiotic signatures in a previously understudied population. Our results underscore the potential of dual-compartment microbiome profiling for developing non-invasive biomarkers and require validation in larger, multicenter cohorts to elucidate mechanistic links between respiratory dysbiosis and colorectal carcinogenesis.

## 1. Introduction

Colorectal cancer (CRC) imposes a considerable economic and healthcare burden worldwide due to its high incidence and frequent late-stage diagnosis. CRC is the third-most commonly diagnosed cancer, following breast and lung cancers, and accounts for more than 700,000 deaths annually [1,2]. These figures underscore the growing focus on identifying new etiological factors of CRC pathogenesis. The role of the gut microbiota in tumor initiation and progression is well established across various malignancies, particularly gastrointestinal (GI) cancers. The GI tract is extensively colonized by a dynamic microbial community [3].

Gut microbial dysbiosis may result from chronic inflammation, yet it can also function as an independent driver of sustained inflammatory responses and carcinogenesis [3]. Studies across diverse populations have linked specific colonic bacterial taxa to CRC occurrence [4,5,6,7,8]. To date, several bacterial species are identified as CRC-associated and exhibit oncogenic potential, as demonstrated in vivo. These include *Bacteroides fragilis, Fusobacterium nucleatum*, and others [9]. However, no single bacterial species has been validated as a universal CRC biomarker. This limitation likely reflects underlying ethnogeographic heterogeneity. Notably, *Fusobacterium nucleatum* remains consistently detectable in CRC specimens in the majority of cases. Previous studies investigating gut microbiota associations with CRC have predominantly focused on North American, European, and Asian cohorts, revealing substantial compositional variations driven by geographic, ethnic, and demographic factors [10,11].

Emerging evidence suggests a ‘gut–lung axis’ whereby gastrointestinal dysbiosis can influence respiratory immune homeostasis, and vice versa [12]. However, whether CRC-associated gut microbial shifts are reflected in the respiratory microbiota remains unknown. Characterizing the sputum microbiome in CRC patients may reveal novel microbial signatures.

The goal of this observational study was to concurrently characterize the bacterial composition of the gut and respiratory microbiomes in CRC patients residing in Western Siberia, a distinct region of Russia characterized by specific environmental exposures and unique dietary patterns. We examined the taxonomic composition and gut–respiratory microbiome interplay in CRC patients.

## 2. Methods

### 2.1. Characteristics of the Studied Groups

The bacterial composition of the gut and sputum microbiomes was analyzed in 40 patients with histopathologically confirmed CRC (18 men, 22 women; mean age: 66.5 ± 8.23 years). All patients were newly diagnosed and presented for initial clinical evaluation at the Kemerovo Regional Oncological Dispensary (Kemerovo, Russian Federation). The control group comprised 45 healthy volunteers (15 men, 30 women; mean age: 57.6 ± 9.71 years) with no personal history of malignancy. Data collection was conducted between May and October of 2025. For each study participant, a standardized questionnaire was administered to capture demographic and clinical variables, including place and date of birth, residence type (urban/rural), history of chronic diseases, current medication use, dietary patterns, prior radiological procedures, smoking status, and alcohol consumption. For CRC patients, additional data were extracted from clinical and histopathological records, including disease stage according to the TNM (tumor, node, metastasis) classification [13].

Inclusion criteria comprised an age of ≥40 years for both sexes, the ability to provide adequate fecal and sputum specimens, and the provision of written informed consent. For CRC patients, eligibility additionally required the absence of surgical intervention or chemoradiotherapy prior to sample collection. Exclusion criteria included a diagnosis of inflammatory bowel disease (IBD), antibiotic use within the preceding six months, colonoscopy performed within one month prior to enrollment, inability to collect sufficient fecal or sputum samples, or refusal to provide informed consent. All participants were fully informed about the study objectives and potential risks and provided written informed consent. The study protocol was approved by the Ethics Committee of Kemerovo State University (Protocol No. 16, dated 9 September 2025). All procedures involving human participants were conducted in accordance with the ethical principles of the Declaration of Helsinki of the World Medical Association (1964, revised 2000).

### 2.2. Collection and Storage of Fecal and Sputum Samples

Fresh fecal (≥4 g) and sputum (2–3 mL) samples were collected from CRC patients and control participants to characterize the taxonomic composition of the gut and respiratory microbiomes. All specimens were obtained prior to the initiation of any therapeutic interventions and were verified to be free from contamination with urine or toilet water. Sputum and stool samples from each study participant were collected simultaneously in the morning hours (8:00–10:00AM). Spontaneously expectorated sputum was collected after rinsing the mouth three times. Giemsa-stained cytological slide microscopy was used to test random sputum samples and the presence of columnar airway epithelial cells was confirmed. Immediately after collection, samples were transferred into sterile plastic containers, frozen at −20 °C, and transported to the laboratory. Specimens were subsequently stored at −80 °C until bacterial DNA extraction.

### 2.3. DNA Extraction, 16S rRNA Amplification, and Sequencing

Prokaryotic DNA from fecal samples was extracted using the FastDNA TM Spin Kit for Feces (MP Biomedicals, Santa Ana, CA, USA) according to the manufacturer’s instructions. Genomic DNA from sputum samples was extracted using the QIAamp DNA Microbiome Kit from Qiagen (Hilden, Germany) according to the manufacturer’s instructions. For amplification of the V3-V4 region of the 16S rRNA gene, 50 ng of DNA was used.

The methods of 16S rRNA amplification and sequencing were the same as described in our previous study [14]. The V3-V4 region of the 16S rRNA gene was amplified using specific primers (forward primer: 5′-TCGTCGGCAGCGTCAGATGTGTATAAGAGACAGCCTACGGGNGGCWGCAG-3′; reverse primer: 5′-GTCTCGTGGGCTCGGAGATGTGTATAAGAGACAGGACTACHVGGGTATCTAATCC-3′) with BioMaster Hi-Fi LR 2X ReadyMix DNA polymerase (Biolab-Mix, Novosibirsk, Russia). Cycling conditions: 94 °C (3 min 30 s), followed by 25 cycles of 94 °C (30 s), 55 °C (30 s), 68 °C (40 s), and final elongation at 68 °C (5 min). Libraries were purified using Agencourt AMPure XP beads (Beckman Coulter, Brea, CA, USA) according to the Illumina 16S metagenomic sequencing library protocol. Dual indices and Illumina sequencing adapters from the Illumina Nextera XT Index Kits v2 B and C (Illumina, San Diego, CA, USA) were added to the target amplicons in a second PCR step using BioMaster Hi-Fi LR 2 ReadyMix DNA polymerase (Biolab-Mix, Russia). Library quality was assessed using the Qubit 2.0 fluorometer (Thermo Scientific, Waltham, MA, USA) and the Agilent Bioanalyzer 2100 system (Agilent Technologies, San Diego, CA, USA). Molarity was then adjusted to 4 nM, libraries were denatured and diluted to a final concentration of 8 pM with 10% PhiX buffer for sequencing on the Illumina MiSeq platform (600 cycles) according to the manufacturer’s instructions.

### 2.4. Microbiota Analysis

Primary processing of sequencing data was performed in the QIIME 2 open-source environment [15] (version 2026.4). Read files in the Illumina Casava 1.8 format were imported and subjected to denoising using the DADA2 algorithm. This yielded amplicon sequence variants (ASVs), a feature table, and a set of representative sequences. Truncation and trimming parameters for the reads were determined individually based on visual assessment of quality profiles. To minimize the impact of background contamination, the frequency method of the decontam algorithm was applied. Due to the absence of direct DNA concentration measurements, a surrogate metadata table was generated using a custom Python 3.14 script, in which the total number of reads per sample was used as a proxy variable. At the final stage, all ASVs with an estimated contamination probability of *p* ≤ 0.1 were removed from the feature table and the set of representative sequences.

Taxonomic identification of the filtered ASVs was carried out using the Naive Bayes classifier implemented in the feature-classifier module of QIIME 2. Classification was performed using pre-trained models based on reference databases [SILVA (v138.2), Greengenes 2 (v2024-09), and GTDB (v232)].

For phylogenetic analysis, multiple alignment of representative sequences (ASVs) was performed using the MAFFT algorithm. Based on the aligned sequences, a rooted phylogenetic tree was constructed using the maximum likelihood method (FastTree). Assessment of alpha and beta diversity of microbial communities was carried out after rarefaction (normalization) to 4000 reads per sample. The adequacy of the chosen sequencing depth for representative description of diversity was confirmed by rarefaction curve analysis. Alpha diversity metrics calculated included the Shannon index, Faith’s phylogenetic diversity (Faith’s PD), and Pielou’s evenness. A set of beta diversity metrics was applied: those accounting for phylogenetic relatedness (weighted and unweighted UniFrac), as well as those based solely on presence/absence and relative abundance of taxa (Jaccard and Bray–Curtis). Statistical significance of differences in alpha diversity between sample groups was assessed using the Kruskal–Wallis test. The effect of experimental factors on beta diversity structure was analyzed using permutational multivariate analysis of variance (PERMANOVA).

To identify taxa with significantly different abundances between groups, two complementary approaches were applied. First, we employed the ANCOM-BC2 (Analysis of Compositions of Microbiomes with Bias Correction), which corrects for systematic biases associated with incomplete taxon sampling and allows estimation of absolute abundance changes with adjustment for multiple testing. Second, relative abundances of bacterial taxa were compared using the Mann–Whitney U test following False Discovery Rate (FDR) correction.

### 2.5. Statistical Analysis

Statistical analysis was performed using STATISTICA 10 software (StatSoft, USA). Continuous variables were summarized as mean values (M). Between-group comparisons for continuous variables and relative abundances of bacterial taxa were conducted using the Mann–Whitney U test. Differences were considered statistically significant at *p* < 0.05. To control for multiple testing, *p*-values were adjusted using the FDR correction. Correlation analysis was performed using Spearman’s rank correlation coefficient.

Associations between the identified microorganisms and comorbidities were assessed using the Kruskal–Wallis test followed by Dunn’s post hoc pairwise test. Correction for multiple comparisons was performed using the Benjamini–Hochberg method; results were considered statistically significant at an adjusted *p* < 0.05.

## 3. Results

### 3.1. Comparison of Clinical and Demographic Characteristics Between Healthy Controls and CRC Patients

To characterize the gut and respiratory microbiomes, fecal and sputum samples were collected from 40 CRC patients and 45 healthy controls. Demographic and clinicopathological characteristics of the study participants are summarized in Table 1. The mean age was significantly higher in the CRC group compared to controls (66.5 vs. 57.6 years, *p* = 0.01). No significant differences were observed between groups with respect to sex distribution or residence type. Regarding smoking status, 33.3% of controls were classified as current smokers and 66.7% as non-smokers. In contrast, among CRC patients, only 3 individuals (9.8%) were current smokers, whereas 90.2% were non-smokers.

Expectedly, cancer patients had a higher prevalence of comorbidities than controls, including cardiovascular diseases, type 2 diabetes mellitus, and obesity (*p* < 0.05, Table 1). The most frequently recorded conditions in healthy donors were cardiovascular diseases (26.7%) and gastric disorders (26.7%).

Among the cancer patient cohort, histologically confirmed diagnoses comprised 92.5% adenocarcinoma and 7.5% other types (signet-ring cell carcinoma, carcinoma in situ within a villous adenoma). By TNM classification, 30% of patients presented with stage 0–I disease, 37.5% with stage II, 25% with stage III, and 7.5% with stage IV.

### 3.2. Gut Microbiome in CRC Patients and Healthy Controls

We sequenced the V3-V4 hypervariable region of the bacterial 16S rRNA gene in fecal samples from 36 CRC patients (9 subjects could not produce a fecal sample) and 45 controls. After excluding these 9 subjects, CRC patients were still on average older, demonstrated less smoking and alcohol consumption, and had a higher incidence of cardiovascular disease and obesity (Supplementary Table S1).

Sequencing identified thirteen phyla, with Firmicutes and Bacteroidetes collectively accounting for over 70% of sequences (Supplementary Figure S1A). A total of 171 genera were detected in the gut microbiomes yet only 15 had a relative abundance > 1% (Supplementary Table S2).

**Figure 1:**
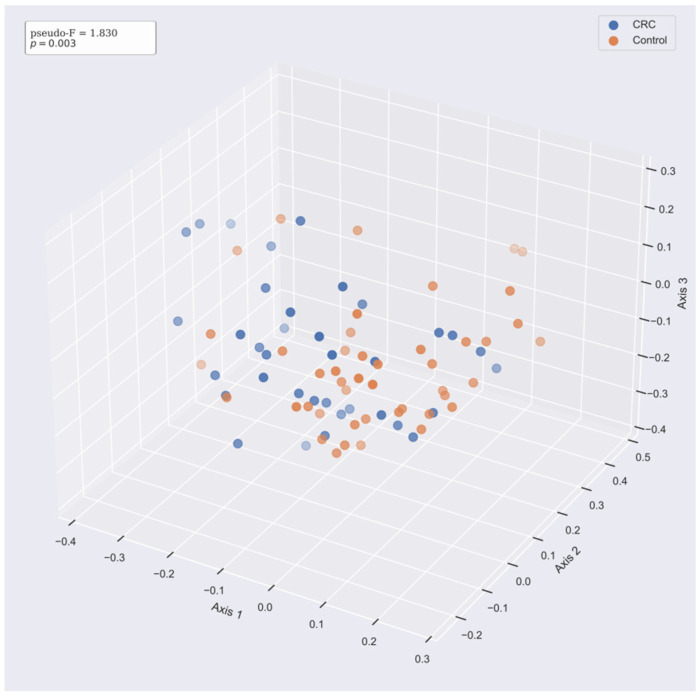
Three-dimensional diagram constructed by principal component analysis showing the phylogenetic diversity of prokaryotic communities in the gut of colorectal cancer patients and control donors (Bray–Curtis, pseudo-F = 1.83; *p* = 0.003).

Gut microbiomes of CRC patients and controls showed no significant differences in alpha-diversity based on Shannon (H = 1.57; *p* = 0.20), Faith’s phylogenetic diversity (Faith’s PD: H = 0.37; *p* = 0.54), or Pielou’s evenness (H = 0.59; *p* = 0.44) indices (Supplementary Figure S2). In contrast, beta-diversity analysis assessed by PERMANOVA (Adonis) using a Bray–Curtis dissimilarity matrix revealed statistically significant differences in microbial composition between CRC patients and healthy controls (Figure 1).

**Figure 2:**
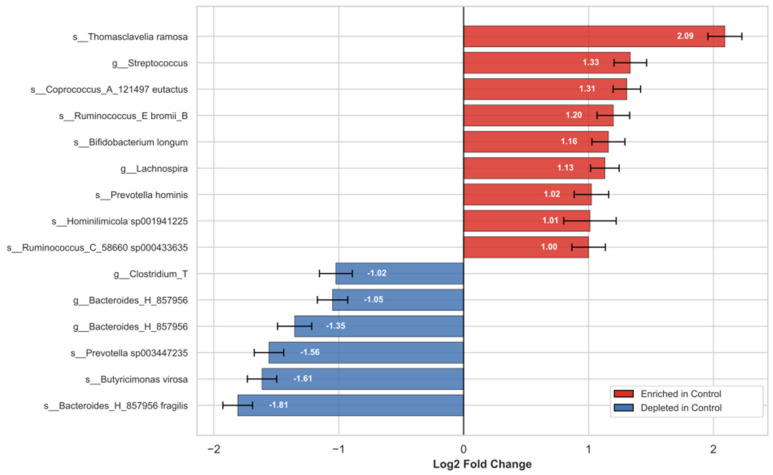
Differentially represented gut microbiome taxa between CRC patients and healthy controls, based on the ANCOM-BC2 method. The diagram shows log2 fold change values for taxa exhibiting statistically significant differences between CRC patients and healthy controls (adjusted for multiple testing, *p*-value < [0.05]).

Differences in bacterial taxonomic composition between CRC and control samples were analyzed using ANCOM-BC2. As shown in Figure 2, the gut microbiome of CRC patients was enriched in the *Bacteroides* (s_ *Bacteroides_H_857956 fragilis*), *Butyricimonas virosa*, *Prevotella sp003447235* and *Clostridium*. Conversely, *Thomasclavelia ramosa*, *Streptococcus*, *Coprococcus*_A_121497 *eutactus*, *Ruminococcus* (*Ruminococcus*_E *bromii*_B and *Ruminococcus*_*C_58660 sp000433635*), *Bifidobacterium longum*, *Lachnospira*, *Prevtella hominis* and *Hominilimicola* sp001941225 were significantly depleted.

We also used an alternative complimentary approach by pairwise comparison of gut microbiota between CRC patients and healthy controls using the non-parametric Mann–Whitney U test (Table 2 and Table 3). The gut microbiome of CRC patients exhibited significant enrichment of the phyla *Proteobacteria* (6.88% vs. 5.44%; *p* = 0.02) and *Fusobacteria* (0.48% vs. 0.14%; *p* = 0.0002), as well as the genera *Bacteroides* (17.19% vs. 12.1%; *p* = 0.025), *Fusobacterium* (0.48% vs. 0.14%; *p* = 0.0002), *Odoribacter* (0.50% vs. 0.19%; *p* = 0.0001), *Sellimonas* (0.93% vs. 0.63%; *p* = 0.0001), *Lachnospiraceae* UCG-010 (0.30% vs. 0.12%; *p* = 0.002), and *Parvimonas* (0.30% vs. 0.12%; *p* = 0.002). Conversely, a significant depletion was observed for the genera *Ruminococcus*, *Incertae Sedis*, *Lachnospira*, *Fusicatenibacter*, and *Cloacibacillus* in CRC patients (Table 3).

To assess potential associations between bacterial taxon abundance and age, Spearman’s rank correlation analysis was performed. The abundance of *Streptococcus* showed a positive correlation with age in healthy controls (r = 0.3162; *p* = 0.0343), but not in CRC patients. No significant differences in the relative abundance of any bacterial genus were detected between males and females within the CRC group. However, in the control group, *Faecalibacterium* was significantly more abundant in males than in females (8.04% vs. 5.09%; *p* = 0.01), while *Dickeya* was significantly more abundant in healthy females compared to males (3.62% vs. 1.03%; *p* = 0.01).

The role of tobacco smoking in shaping the gut microbiome structure was only evaluated in the control group because only three CRC patients were smokers. Smokers exhibited significantly higher relative abundance of *Incertae Sedis* (family *Ruminococcaceae*) compared to non-smokers (0.45% vs. 0.24%; *p* = 0.02). Alcohol consumption was only associated with reduced abundance of *Lachnospira* in CRC patients (0.09% vs. 0.83%; *p* = 0.01), but not in healthy donors. Kruskal–Wallis test revealed no significant associations between comorbidities and bacterial taxa levels in the stool of patients or in the stool of the control group (*p* < 0.05).

Gut microbiota composition differed minimally between CRC patients who adhered to a prescribed diet and those who did not. The only significant difference was a lower relative abundance of the genus *UCG-003* (family Oscillospiraceae) in diet-adherent patients (0.07% vs. 0.36%; *p* = 0.01).

Disease stages among enrolled CRC patients varied according to the TNM classification (Table 1), so we compared bacterial taxon abundances in patients with early-stage disease (stages I-II) versus advanced-stage disease (stages III-IV). Only the genus *Akkermansia* was significantly more abundant in fecal samples from patients with advanced CRC (0.85% vs. 2.24%; *p* = 0.02), a finding corroborated at the phylum level for *Verrucomicrobiota*, to which *Akkermansia* belongs (0.89% vs. 2.31%; *p* = 0.02).

### 3.3. Sputum Microbiome in CRC Patients Versus Healthy Controls

**Figure 3:**
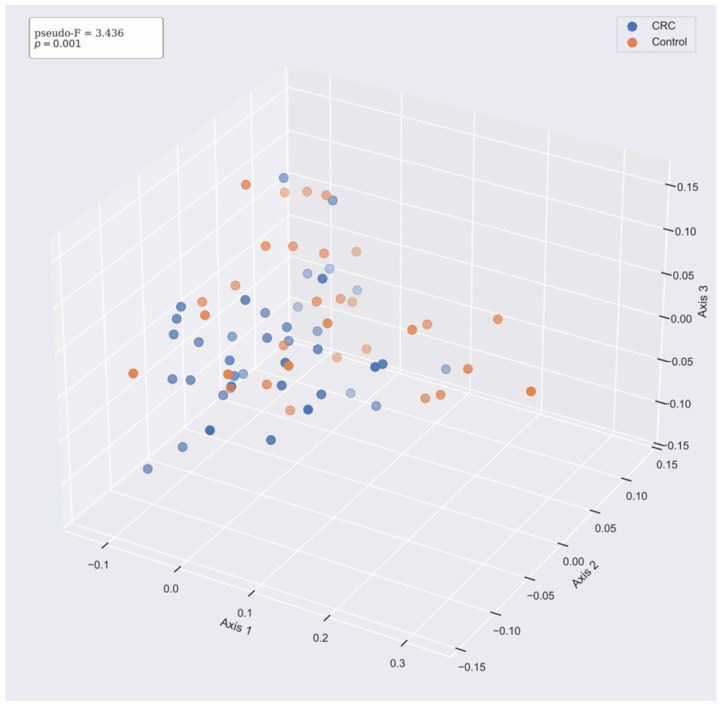
Three-dimensional diagram constructed by principal component analysis showing the phylogenetic diversity of prokaryotic communities in the sputum of colorectal cancer patients and control donors (Weighted UniFrac, pseudo-F = 3.43; *p* = 0.001).

We analyzed sputum samples from 39 CRC patients and 33 healthy controls, as 12 individuals could not produce sufficient sputum. After excluding these missing samples, subjects with CRC were on average older, demonstrated less pronounced smoking and drinking habits, but had a higher incidence of cardiovascular disease, diabetes, urogenital diseases, and other conditions (Supplementary Table S3). A total of 15 bacterial phyla were identified, with *Bacteroidetes* and *Firmicutes* being the most abundant (Supplementary Figure S1B). The relative abundances of 64 bacterial genera detected in sputum samples across study participants are summarized in Supplementary Table S4. Respiratory tract (sputum) microbiomes of CRC patients and controls showed no significant differences in alpha-diversity (Supplementary Figure S3). In contrast, beta-diversity analysis revealed statistically significant differences in microbial community composition between CRC patients and healthy controls (Figure 3).

Application of univariate non-parametric statistics (Mann–Whitney U test) identified bacterial taxa with differential abundance in sputum samples from CRC patients versus healthy controls. CRC patients exhibited significantly higher relative abundances of the following phyla: *Bacteroidetes* (32.76% vs. 26.92%; *p* = 0.005), *Actinobacteria* (10.3% vs. 8.28%; *p* = 0.01), and *Campylobacterota* (1.81% vs. 1.29%; *p* = 0.01) compared to controls (Table 4). At the genus level (Table 5), sputum samples from cancer patients showed significant enrichment of *Nocardia* (9.67% vs. 7.69%; *p* = 0.005), *Leptotrichia* (1.92% vs. 1.24%; *p* = 0.006), *Campylobacter* (1.79% vs. 1.28%; *p* = 0.01), and the *Eubacterium nodatum* group (0.21% vs. 0.09%; *p* = 0.005). Conversely, the relative abundances of five genera, namely *Treponema*, *Oribacterium*, *Pseudomonas*, *Porphyromonas*, and *Saccharimonadales*, were significantly lower in sputum from CRC patients in comparison with (Table 5).

**Figure 4:**
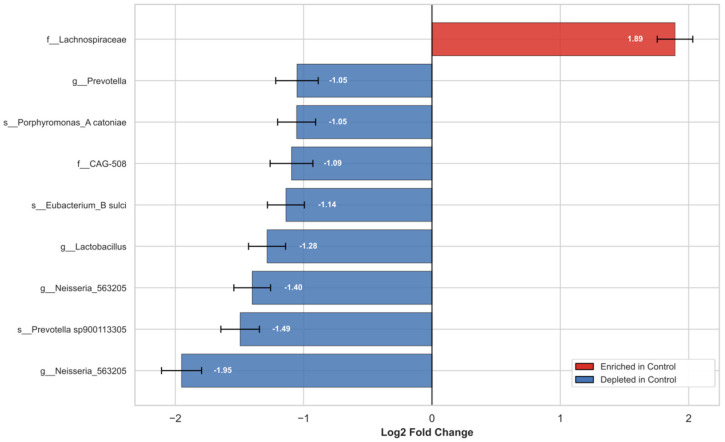
Differentially represented microbiome taxa between CRC patients and healthy controls, performed using the ANCOM-BC2 method. The diagram displays the log2 fold change values for taxa showing statistically significant differences between the CRC group and the healthy control reference group (adjusted for multiple testing, *p*-value < [0.05]).

ANCOM-BC2 analysis also identified differences in the abundance of bacterial taxa in the respiratory tract of CRC patients compared to controls (Figure 4). Specifically, sputum samples from CRC patients were enriched in *Neisseria*, *Prevotella*, *Lactobacillus*, *Eubacterium_B sulci* and *Porphyromonas_A catoniae*. CRC patients also exhibited increased abundances of the CAG-508 family (*Clostridium* sp. *CAG*:508).

Conversely, sputum from healthy subjects exhibited higher relative abundances of the *Lachnospiraceae*.

Spearman’s rank correlation analysis revealed a positive association between age and the abundance of *Bacteroides* in sputum from CRC patients (r = 0.4222; *p* = 0.0074), and an inverse correlation with *Porphyromonas* (r = −0.3515; *p* = 0.0282). Distinct age-related correlations were observed in sputum from healthy controls: *Lactobacillus* abundance increased with age (r = 0.4477; *p* = 0.009), whereas *Butyrivibrio* and *Catonella* showed negative correlations with age (r = −0.3988; *p* = 0.0215 and r = −0.3711; *p* = 0.0335).

No significant differences in the relative abundance of any bacterial genus were detected between males and females in the control group. However, among CRC patients, *Fusobacterium* was significantly more abundant in females than in males (3.91% vs. 2.85%; *p* = 0.02). In sputum from controls who smoked, three genera showed significantly lower abundance compared to non-smokers: *Tepidibacter* (0.05% vs. 0.42%; *p* = 0.01), *Eubacterium nodatum* group (0.06% vs. 0.11%; *p* = 0.004), and *Catonella* (0.04% vs. 0.34%; *p* = 0.01). Alcohol consumption and dietary adherence showed no significant associations with bacterial abundance in sputum from either CRC patients or controls. Using Kruskal–Wallis test followed by Dunn’s post hoc pairwise test, significant associations were found between cardiovascular diseases in CRC patients with the presence of Leptotrichia and Helicobacter in the sputum, whereas Lachnoanaerobaculum was associated with liver disease. However, these correlations were not observed in healthy controls.

The CRC stage was associated with the abundance of certain bacterial taxa in sputum. The phylum *Campylobacterota* was more abundant in early-stage disease (I–II) compared to advanced-stage disease (III–IV) (2.08% vs. 1.33%; *p* = 0.02), a pattern mirrored at the genus level for *Campylobacter* (2.06% vs. 1.30%; *p* = 0.01). Conversely, *Abiotrophia* was significantly more abundant in advanced-stage CRC compared to early-stage disease (0.16% vs. 0.06%; *p* = 0.02).

## 4. Discussion

This report provides the first simultaneous characterization of gut and respiratory microbiota profiles in CRC patients from a Russian cohort of Western Siberia. Whereas taxonomic alterations in the human gut microbiome are widely recognized as a pathogenic factor in CRC [16,17,18,19,20], questions regarding the existence of region-specific microbial signatures associated with the pathogenesis and progression of CRC remain obscure. Inhabitants of Western Siberia consume a Western-type diet high in red meat and low in fiber, which promotes procarcinogenic metabolites [21,22] and reduces protective SCFAs [23]. This dietary pattern likely contributed to the high prevalence of comorbid conditions in our CRC cohort [24].

An unexpected finding was that among the 40 CRC patients, only three were current smokers, as opposed to 35% of healthy controls who reported this habit. This observation appears to contradict the fact that smoking is a recognized risk factor for CRC development [25] and warrants further investigation.

Previous studies of the gut bacterial microbiota in CRC often show a decrease in the alpha-diversity of the intestinal microflora [26,27,28]. Our study demonstrated that alpha-diversity indices (Shannon, Pielou’s evenness, and Faith’s PD) did not differ significantly between CRC patients and controls, in either gut or respiratory microbiomes. In contrast, beta-diversity analysis revealed substantial compositional differences between groups for both fecal and sputum samples. These findings regarding alpha- and beta-diversity of gut microbial communities are in good agreement with recent results obtained from large-scale cohorts of CRC patients and controls in Korea [29]. We were unable to compare our findings on microbiota diversity in sputum samples from CRC patients and healthy individuals with results from other studies, as no comparable investigations have been reported to date. Unlike our sputum findings, a prior study of the oral microbiome in CRC reported higher alpha-diversity in patients [30].

The taxonomic differences in gut and respiratory microbiota between CRC patients and healthy controls observed in our study are corroborated by the differential abundance of specific bacterial taxa across multiple taxonomic levels, as identified through univariate statistical analysis and ANCOM-BC2. For clarity, all statistically significant differences are summarized in Table 6.

Fecal and sputum samples exhibited distinct taxonomic signatures, with different bacterial groups showing significant differential abundance between CRC patients and controls. In the gut microbiome of CRC patients, we observed a significant enrichment of the phyla *Proteobacteria* and *Fusobacteria*, which aligns with previously reported findings [8,19,27,29]. Pathogenic mechanisms of *Fusobacterium nucleatum* in CRC development have been extensively studied [31,32,33,34].

Representatives of *Bacteroides* were also observed in greater abundance in the gut of patients compared to controls. In this regard, the effects of enterotoxigenic *Bacteroides fragilis* (ETBF) are well known, as they play a significant role in CRC development [35]. According to our data, the relative abundance of several other bacterial genera was significantly higher in CRC patients compared to controls, including *Odoribacter* (0.5% vs. 0.18%; *p* = 0.003), *Lachnospiraceae UCG-010* (0.3% vs. 0.12%; *p* = 0.002)*, Erysipelatoclostridium* (0.08% vs. 0.05%; *p* = 0.004), *Parvimonas* (0.08% vs. 0.0001%; *p* = 0.001), and *Finegoldia* (0.06% vs. 0.0005%; *p* = 0.007). Some of these taxa have been previously discussed in the context of CRC. For example, *Odoribacter* was enriched in a murine model of CRC (ApcMin/+) [36], whereas *Parvimonas spp.* was overrepresented in the gut microbiome of CRC patients [37].

Sex, age, smoking, and alcohol consumption did not affect the gut microbiome composition of CRC patients (Mann–Whitney U test), suggesting that pathogenic processes exert a predominant influence on the composition of the intestinal microbiota. The only exception was a significant reduction in bacteria of the genus *UCG-003* (family *Oscillospiraceae*, class *Clostridia*) in the stool of CRC patients adhering to a prescribed diet. Notably, the role of these bacteria has been described in both positive and negative contexts, they are recognized as producers of short-chain fatty acids known to stimulate immune system function [38], yet they have also been included among bacterial taxa associated with depression [39].

Alongside the gut microbiome, analysis of the bacterial microbiota in the lower respiratory tract also yielded several noteworthy findings in the context of CRC. The enrichment of *Neisseria*, *Nocardia*, *Leptotrichia*, *Campylobacter*, and the *Eubacterium nodatum group* detected in the sputum microbiome of CRC patients supports the establishment of a pathogenic microbiota within the patients’ respiratory tract. Bacteria belonging to the genus *Nocardia* are known to cause severe systemic bacterial infections, attributable in part to their intrinsic resistance to beta-lactam antibiotics [40]. Bacteria of the genera *Leptotrichia* and *Campylobacter* have even been proposed as biomarkers for screening populations to detect CRC [41]. Furthermore, an increase in the abundance of *Eubacterium nodatum* bacteria induced CRC progression in mice [42]. Recently, an increase in the abundance of *Rothia* was found in the saliva of CRC patients [43].

*Helicobacter* was enriched in sputum of CRC patients. Although *H. pylori* infection is associated with increased CRC risk [44,45,46], this association has previously been confined to the gut. Detection of *Helicobacter* in sputum may reflect oral contamination [47], as the oral cavity can harbor these bacteria. Alternatively, respiratory colonization cannot be ruled out and requires further study.

As observed in the gut microbiome, the respiratory microbiome of CRC patients exhibited a reduced abundance of the genera *Treponema*, *Oribacterium*, *Pseudomonas*, *Porphyromonas*, *Saccharimonadales*, and *Shuttleworthia* compared to controls. The potential protective role of these bacteria in mitigating CRC risk remains to be investigated. Nevertheless, the reduced abundance of *Treponema* in the sputum of CRC patients is consistent with recently published data suggesting a protective effect of these taxa. It has even been proposed that *Treponema* levels in the saliva of CRC patients may provide sufficient predictive accuracy for CRC development [48]. The abundance of *Oribacterium* in the feces of CRC patients was associated with early stages of the tumor process [49]. Although the pathogenic potential of bacteria belonging to the genus *Pseudomonas* has been well established, recent evidence has also demonstrated that these bacteria are capable of activating adaptive immunity in the context of CRC [50].

Several limitations of this study should be acknowledged. First, the sample size (81 stool and 73 sputum samples) may be insufficient to ensure representative findings. Additionally, there is imbalance between the study groups in terms of age and comorbidities, with a tendency toward imbalance in gender, diet, and smoking habits as well. Second, we used 16S rRNA gene amplicon sequencing for microbiome analysis rather than shotgun metagenomics, which would provide higher resolution and enable species-level identification. This limitation prevented us from drawing conclusions about potential interactions between the intestinal and respiratory microbiota in these patients at this stage. Third, sputum collection carries the risk of contamination with oral microbiota, so we cannot rule out the possibility of such contamination.

In conclusion, our observational data underscore the promising potential of concurrently investigating the gut and respiratory tract microbiomes in the context of CRC. This area of research is gaining increasing attention for its potential to enable early, non-invasive CRC detection and its prospective utility in therapeutic applications [51].

## Figures and Tables

**Table 1:** Clinicopathological characteristics of the study groups.

Baseline Characteristics	CRC Patients, n = 40	Healthy Individuals, n = 45
Age, years/Min–Max	66.5 */43–84	57.6/40–77
Gender (%):		
Male	45.0	33.3
Female	55.0	66.7
Place of residence (%):		
City	82.5	75.6
Village	17.5	24.4
Diet (%):		
Yes	35.0 *	2.2
No	65.0	97.8
Smoking (%):		
Yes	7.5 *	33.3
No	92.5	66.7
Alcohol consumption (%):		
Yes	62.5	62.2
No	37.5	37.8
Chronic conditions (%):		
Cardiovascular disease	87.5 *	26.7
Bronchitis, COPD	5.0	11.1
Stomach	27.5	26.7
Diabetes	17.5 *	2.2
Obesity	20.0 *	0
Liver	5.0	6.7
Urogenital	17.5	6.7
Blood	12.5	2.2
Histological subtype of CRC (%):		-
Adenocarcinoma	92.5	
Others	7.5	
TNM ^#^ (%):		-
0–I	30.0	
II	37.5	
III	25.0	
IV	7.5	

Abbreviations: COPD, Chronic obstructive pulmonary disease; TNM, Tumor, node, metastasis. Mann–Whitney U test was used for comparisons, * Significantly different vs control group, ^#^ Tumor, Node, Metastasis.

**Table 2:** Average percentage of bacterial phyla in the gut microbiomes of CRC patients and healthy donors.

Phyla	CRC	Control	*p*
*Firmicutes*	46.48	50.18	>0.05
*Bacteroidetes*	31.49	28.1	>0.05
*Actinobacteria*	6.22	6.87	>0.05
*Proteobacteria*	6.88 ↑	5.44	0.02
*Verrucomicrobia*	1.38	1.25	>0.05
*Fusobacteria*	0.48 ↑	0.14	0.0002
*Spirochaetes*	0.003	0.01	>0.05
*Synergistes*	0.006	0.05	>0.05
*Cyanobacteria*	0.003	0.03	>0.05
*Lentisphaerae*	0.009	0.009	>0.05
*Campylobacterota*	0.009	0.03	>0.05
*Desulfobacterota*	0.009	0.007	>0.05
*Patescibacteria*	0.005	0.03	>0.05

↑—increase compared to the value for the Controls.

**Table 3:** Average percentage of bacterial genera in the gut microbiomes of CRC patients and healthy donors.

Genus	CRC	Control	*p*
*Bacteroides*	17.19 ↑	12.1	0.025
*Odoribacter*	0.5 ↑	0.18	0.003 *
*Fusobacterium*	0.48 ↑	0.14	0.0002 *
*Lachnospiraceae_UCG-010*	0.3 ↑	0.12	0.002 *
*Erysipelatoclostridium*	0.08 ↑	0.05	0.004 *
*Parvimonas*	0.08 ↑	0.0001	0.001 *
*Peptoniphilus*	0.008 ↑	0.0002	0.03
*Staphylococcus*	0.005 ↑	0.0001	0.04
*Finegoldia*	0.006 ↑	0.0005	0.007 *
*Incertae Sedis*	0.24 ↓	0.31	0.01 *
*Clostridia_UCG-014*	0.21 ↓	0.38	0.04
*Fusicatenibacter*	0.19 ↓	0.43	0.004 *
*Monoglobus*	0.17 ↓	0.28	0.05
*Ruminococcus (f. Ruminococcaceae)*	0.16 ↓	0.51	0.008 *
*Lachnospira*	0.16 ↓	0.41	0.01 *
*Cloacibacillus*	0.002 ↓	0.05	0.008 *
*Eggerthella*	0.007 ↓	0.07	0.001 *

↑—increase compared to the value for the Controls; ↓—decrease compared to the value for the Controls. * Significant after false discovery rate (FDR) correction.

**Table 4:** Average percentage of bacterial phyla in the sputum microbiomes of CRC patients and healthy donors.

Phyla	CRC	Control	*p*
*Bacteroidetes*	32.76 ↑	26.92	0.005 *
*Firmicutes*	23.03	29.64	>0.05
*Proteobacteria*	14.53	14.99	>0.05
*Actinobacteria*	10.3 ↑	8.28	0.01 *
*Fusobacteria*	5.84	6.8	>0.05
*Patescibacteria*	2.88	2.12	>0.05
*Campylobacterota*	1.81 ↑	1.29	0.01 *
*Spirochaetes*	0.91	1.57	>0.05
*Synergistes*	0.12	0.19	>0.05
*Cyanobacteria*	0.002 ↓	0.01	0.01 *
*Verrucomicrobia*	0.001	0.001	>0.05
*SR1*	0.001	0.0001	>0.05
*Chloroflexi*	0.0002 ↓	0.004	0.01 *
*Desulfobacterota*	0	0.0003	>0.05
*TM7*	0.0001	0	>0.05

↑—increase compared to the value for the Controls; ↓—decrease compared to the value for the Controls. * Significant after false discovery rate (FDR) correction.

**Table 5:** Average percentage of bacterial genera in the sputum microbiomes of CRC patients and healthy donors.

Genus	CRC	Control	*p*
*Nocardia*	9.67 ↑	7.69	0.005 *
*Leptotrichia*	1.92 ↑	1.24	0.006 *
*Campylobacter*	1.79 ↑	1.28	0.01 *
*Helicobacter*	1.15 ↑	0.64	0.0001 *
*Rothia*	1.05 ↑	0.53	0.005 *
*[Eubacterium]_nodatum_group*	0.21 ↑	0.09	0.005 *
*Lachnoanaerobaculum*	0.48 ↑	0.22	0.01 *
*Treponema*	0.91 ↓	1.54	0.003 *
*Oribacterium*	0.59 ↓	0.67	0.0002 *
*Pseudomonas*	0.43 ↓	1.36	0.01 *
*Porphyromonas*	0.56 ↓	1.41	0.03
*Saccharimonadales*	0.08 ↓	0.23	0.008 *

↑—increase compared to the value for the Controls; ↓—decrease compared to the value for the Controls. * Significant after false discovery rate (FDR) correction.

**Table 6:** Bacterial taxa with differential abundance in feces and sputum of CRC patients compared with controls.

Taxonomic Level	Feces	Sputum
Phylum	*Proteobacteria* ↑, *Fusobacteria* ↑	*Bacteroidetes* ↑, *Actinobacteria* ↑, *Campylobacterota* ↑; *Cyanobacteria* ↓, *Chloroflexi* ↓
Family	-	*Clostridium* sp. *CAG*:508 ↑ *Lachnospiraceae* ↓
Genus	*Fusobacterium* ↑*,* *Bacteroides* ↑*, Odoribacter* ↑*, Lachnospiraceae_UCG-010* ↑, *Erysipelatoclostridium* ↑, *Parvimonas* ↑, *Finegoldia* ↑, *Clostridium* ↑ *Streptococcus* ↓, *Incertae Sedis* ↓, *Fusicatenibacter* ↓, *Ruminococcus* ↓, *Lachnospira* ↓, *Cloacibacillus* ↓, *Eggerthella* ↓	*Neisseria* ↑, *Prevotella* ↑, *Lactobacillus* ↑, *Nocardia* ↑, *Leptotrichia* ↑, *Campylobacter* ↑, *Helicobacter* ↑, *Rothia* ↑, *[Eubacterium]_nodatum_group* ↑, *Lachnoanaerobaculum* ↑ *Treponema* ↓, *Oribacterium* ↓, *Pseudomonas* ↓, *Porphyromonas* ↓, *Saccharimonadales* ↓
Species	*Bacteroides*_*H_857956 fragilis* ↑, *Butyricimonas virosa* ↑, *Prevotella sp003447235* ↑ *Thomasclavelia ramosa* ↓, *Coprococcus*_A_121497 *eutactus* ↓, *Ruminococcus*_E *bromii*_B ↓, *Ruminococcus*_C_58660 *sp*000433635 ↓, *Bifidobacterium longum* ↓, *Prevtella homini*s ↓, *Hominilimicola sp*001941225 ↓	*Eubacterium_B sulci* ↑, *Porphyromonas_A catoniae* ↑, *Prevotella sp900113305* ↑

↑—increase compared to Controls; ↓—decrease compared to Controls.

## Data Availability

Microbiome sequencing data that support the findings of this study have been deposited in the BioProject under accession code PRJNA1447746. All other data supporting the findings of this study are available from the corresponding author on reasonable request.

## Supplementary Materials

The following supporting information can be downloaded at: https://www.mdpi.com/article/10.3390/microorganisms14071392/s1, Table S1. Clinicopathological characteristics of the study groups (for stool samples). Table S2. Mean percentages of bacterial genera in the gut of CRC patients and healthy donors. Mann-Whitney U test. Table S3. Clinicopathological characteristics of the study groups (for sputum samples). Table S4. Mean percentages of bacterial genera in the sputum of CRC patients and healthy donors. Mann-Whitney U test. Figure S1. Taxonomic phylum-level structure of gut (A) and sputum (B) microbiomes from CRC patients and healthy donors. Figure S2. Alpha diversity of gut microbiota from CRC patients and healthy donors (top to bottom: Shannon diversity index, Pielou’s index, Faith_pd index). Figure S3. Alpha diversity of sputum microbiota from CRC patients and healthy donors (top to bottom: Shannon diversity index, Pielou’s, Faith_pd index).
