# Supplementary material for: Features of the Intestinal and Respiratory Microbiome in Colorectal Cancer Patients in Western Siberia
Source: Microorganisms. 2026 Jun 23;14(7):1392. doi: 10.3390/microorganisms14071392 (PMC13413513; doi:10.3390/microorganisms14071392)
Supplement: Supplementary file 1 [file microorganisms-14-01392-s001.zip › microorganisms-4348812-supplementary.pdf]

Supplementary Material: Features of the intestinal and respiratory microbiome in colorectal cancer patients in Western Siberia

Table S1. Clinicopathological characteristics of the study groups (for stool samples).

Baseline Characteristics	Colorectal cancer patients, n=36	Healthy Individuals, n=45
Age, years / Min - Max	66.5* / 43-84	57.6 / 40-77
Gender (%):		
Male	42.0	31.0
Female	58.0	69.0
Place of residence (%):		
City	80.6	75.6
Village	19.4	24.4
Diet (%):		
Yes	38.9*	2.2
No	61.1	97.8
Smoking (%):		
Yes	8.3*	33.3
No	91.7	66.7
Alcohol consumption (%):		
Yes	58.3*	80.0
No	41.7	20.0
Chronic conditions (%):		
Cardiovascular disease	86.1*	28.9
Bronchitis, COPD	5.6	11.1
Stomach	30.6	26.7
Diabetes	16.7*	2.2
Obesity	19.4*	0
Liver	5.6	8.9
Urogenital	22.2	6.7
Blood	8.3	2.2
Histological subtype of CRC (%):		
Adenocarcinoma	91.7	-
Others	8.3	
TNM# (%):		
0-I	27.8	
II	36.1	-
III	27.8	
IV	8.3	

Abbreviations: COPD, Chronic obstructive pulmonary disease; TNM, Tumor, node, metastasis. Mann–Whitney U test was used for comparisons, * Significantly different vs. controls.

Table S2. Mean percentages of bacterial genera in the gut of CRC patients and healthy donors. Mann-Whitney U test.

Genus	CRC	Control	p
Bacteroides	17.19↑	12.1	0.03
Faecalibacterium	4.75	6.01	> 0.05
Nocardia	3.83	4.22	> 0.05

Dickeya	3.82	2.82	> 0.05
Alistipes	2.81	2.29	> 0.05
[Ruminococcus]_torques_group	2.42	2.47	> 0.05
Prevotella	1.93	3.39	> 0.05
Subdoligranulum	1.51	1.73	> 0.05
Akkermansia	1.33	1.22	> 0.05
Tepidibacter	1.27	1.11	> 0.05
Streptococcus	1.55	1.24	> 0.05
Clostridium (f.Lachnospiraceae)	1.1	1.23	> 0.05
Anaerostipes	1.05	1.15	> 0.05
[Eubacterium]_hallii_group	1.09	1.07	> 0.05
Collinsella	1.03	0.95	> 0.05
UCG-002	0.98	0.76	> 0.05
Roseburia	0.97	0.87	> 0.05
Blautia	0.97	0.78	> 0.05
Sellimonas	0.93	0.63	> 0.05
Dorea	0.93	0.99	> 0.05
Gemmiger	0.92	0.95	> 0.05
Clostridium (f.Peptostreptococcacaea)	0.85	0.94	> 0.05
Christensenellaceae_R-7_group	0.72	0.26	> 0.05
Clostridium (f.Ruminococcaceae)	0.69	1.21	0.005
Clostridium (f.Clostridiaceae)	0.62	0.68	> 0.05
Butyricicoccus	0.51	0.58	> 0.05
Odoribacter	0.5↑	0.18	0.003
Fusobacterium	0.48↑	0.14	0.0002
Coprococcus	0.07	0.45	> 0.05
Lachnospira	0.4	0.44	> 0.05
Shuttleworthia	0.41	0.52	> 0.05
Ruminococcus(f.Lachnospiraceae)	0.37	0.33	> 0.05
Barnesiella	0.33	0.32	> 0.05
UCG-005	0.31	0.28	> 0.05
Lachnospiraceae_UCG-010	0.3↑	0.12	0.002
Slackia	0.29	0.15	> 0.05
[Eubacterium]_siraeum_group	0.27	0.28	> 0.05
UBA1819	0.25	0.07	> 0.05
UCG-003	0.25	0.2	> 0.05
Incertae_Sedis	0.24↓	0.31	0.01
[Eubacterium]_coprostanoligenes_group	0.24	0.15	> 0.05
Senegalimassilia	0.22	0.25	> 0.05
Clostridia_UCG-014	0.21	0.38	0.04
Fusicatenibacter	0.19↓	0.43	0.004
Olsenella	0.18	0.21	> 0.05
Monoglobus	0.17	0.28	0.05
Allorhizobium-Neorhizobium-Pararhizobium-Rhizobium	0.17	0.13	0,03
Ruminococcus (f.Ruminococcaceae)	0.16↓	0.51	0.008
Lachnospira	0.16↓	0.41	0.01
[Eubacterium]_ruminantium_group	0.16	0.17	> 0.05
Oscillibacter	0.12	0.13	> 0.05
Clostridium (f.Erysipelotrichaceae)	0.12	0.08	0.03
Flavonifractor	0.12	0.08	> 0.05
Methanobrevibacter	0.11	0.12	> 0.05
Lactobacillus	0.09	0.04	> 0.05
Erysipelatoclostridium	0.08	0.05	0.004
Parvimonas	0.08	0.0001	0.001
Paraprevotella	0.08	0.08	> 0.05
Coprococcus	0.07	0.45	> 0.05
Bifidobacterium	0.06	0.1	> 0.05
Howardella	0.06	0.06	> 0.05
Paraeggerthella	0.06	0.08	> 0.05
Holdemanella	0.04	0.04	> 0.05

Dialister	0.04	0.05	> 0.05
NK4A214_group	0.04	0.03	> 0.05
NK3B31_group	0.03	0.08	> 0.05
Lachnospiraceae_UCG-004	0.04	0.01	> 0.05
Megasphaera	0.02	0.03	> 0.05
Pseudomonas	0.03	0.002	> 0.05
Colidextribacter	0.03	0.05	> 0.05
Butyrivibrio	0.03	0.14	> 0.05
Pseudomonas	0.03	0.002	> 0.05
Parabacteroides	0.03	0.02	> 0.05
Prevotellaceae_UCG-001	0.004	0.001	> 0.05
Bilophila	0.001	0.001	> 0.05
Cloacibacillus	0.002↓	0.05	0.008
Terrisporobacter	0.01	0.01	> 0.05
Weissella	0.005	0.002	> 0.05
Lactococcus	0.01	0.02	> 0.05
Coprobacter	0.007	0.01	> 0.05
Phascolarctobacterium	0.01	0.02	> 0.05
Gordonibacter	0.006	0.002	> 0.05
Eggerthella	0.007↓	0.07	0.001
Enterococcus	0.006	0.002	> 0.05
Megamonas	0.0004	0.0009	> 0.05
Methanosphaera	0.009	0.009	> 0.05
Fretibacterium	0.004	0.0004	> 0.05
Victivallis	0.009	0.009	> 0.05
Lactococcus	0.009	0.002	> 0.05
Weissella	0.005	0.002	> 0.05
Eubacterium	0.008	0.003	> 0.05
[Eubacterium]_nodatum_group	0.0007	0.002	> 0.05
[Eubacterium]_brachy_group	0.0002	0.00007	> 0.05
Agathobacter	0.004	0.002	> 0.05
[Clostridium]_innocuum_group	0.006	0.006	> 0.05
Clostridium_sensu_stricto_1	0.01	0.05	> 0.05
Anaerofustis	0.001	0.0006	> 0.05
Pedobacter	0.008	0.002	> 0.05
Campylobacter	0.005	0.03	> 0.05
Rothia	0.003	0.01	> 0.05
Porphyromonas	0.003	0.003	> 0.05
Finegoldia	0.006	0.0005	0.007
Selenomonas	0.0003	0.009	> 0.05
Neisseria	0.0002	0.02	> 0.05
Lachnoanaerobaculum	0.0004	0.004	> 0.05
Actinomyces	0.001	0.0004	> 0.05
Comamonas	0.0004	0.0002	> 0.05
Saccharimonadaceae	0.0004	0.002	> 0.05
Streptobacillus	0.0009	0.0001	> 0.05
Peptoniphilus	0.008	0.0002	0.03
Christensenella	0.001	0.001	> 0.05
Catabacter	0.001	0.002	> 0.05
Staphylococcus	0.005	0.0001	0.04
Turicibacter	0.002	0.005	> 0.05
Solobacterium	0.0002	0.0002	> 0.05
Veillonella	0.01	0.001	> 0.05
Leptotrichia	0,001	0.008	> 0.05
Moryella	0.008	0.01	> 0.05
Peptostreptococcus	0.01	0	0.002
Blautia	0.0004	0.0003	> 0.05
Peptococcus	0.001	0.0008	> 0.05
Pediococcus	0.02	0.03	> 0.05
Catenibacterium	0.01	0.03	> 0.05
Erysipelotrichaceae_UCG-003	0.02	0.01	> 0.05
Cytophaga	0.01	0.007	> 0.05

Desulfovibrio	0.005	0.006	> 0.05
Paludicola	0.006	0.003	> 0.05
Treponema	0.003	0.01	> 0.05
Butyricimonas	0.007	0.003	0.002
Bilophila	0.001	0.001	> 0.05
Phascolarctobacterium	0.01	0.02	> 0.05
Catenibacterium	0.01	0.03	> 0.05
Sporobacter	0.008	0.004	> 0.05
Lactonifactor	0.0009	0.001	> 0.05
Colidextribacter	0.03	0.05	> 0.05
Raoultibacter	0.0004	0.001	> 0.05
Anaerotruncus	0.02	0.004	0.02
Anaerofilum	0.002	0.003	> 0.05
Anaerococcus	0.02	0.001	0.005
Listeria	0.002	0.007	> 0.05
Mogibacterium	0.001	0	> 0.05
Tyzzerella	0.001	0.008	> 0.05
Helicobacter	0.0007	0.02	> 0.05
UCG-009	0.005	0.006	> 0.05
UCG-010	0.002	0.005	> 0.05
Libanicoccus	0.03	0.09	> 0.05
Phocea	0.002	0.006	> 0.05
Negativibacillus	0.003	0.006	> 0.05
Paludicola	0.006	0.003	> 0.05
NK4A214_group	0.04	0.03	> 0.05
Family_XIII_AD3011_group	0.04	0.02	> 0.05
Acetanaerobacterium	0.005	0.002	> 0.05
Gastranaerophilales	0.003	0.02	> 0.05
Chloroplast	0	0.001	> 0.05
Catonella	0	0.003	> 0.05
Oribacterium	0	0.004	> 0.05
Parabacteroides	0	0.0001	> 0.05
Stomatobaculum	0	0.002	> 0.05
Capnocytophaga	0	0.0002	> 0.05
Clostridia_vadinBB60_group	0	0.001	> 0.05
Lachnospiraceae_NK4A136_group	0	0.00009	> 0.05
UCG-001	0.0007	0	> 0.05
Sutterella	0.0002	0	> 0.05
Lachnoclostridium	0.007	0	> 0.05
Mycoplasma	0.0001	0	> 0.05
Bulleidia	0.0002	0	> 0.05
Sneathia	0.0002	0	> 0.05
Methanomassiliicoccus	0.0002	0	> 0.05
Filifactor	0.002	0	> 0.05
Escherichia-Shigella	0.0002	0	> 0.05
Pseudoramibacter	0.003	0	> 0.05

↑ - increase compared to the value for the Controls; ↓ - decrease compared to the value for the Controls.

Table S3. Clinicopathological characteristics of the study groups (for sputum samples).

Baseline Characteristics	Colorectal cancer patients, n=39	Healthy Individuals, n=33
Age, years / Min - Max	66.6* / 43-84	55.6 / 40-77
Gender (%):		
Male	43.6	39.4
Female	56.4	60.6
Place of residence (%):		
City	82.0	87.9
Village	18.0	12.1
Diet (%):		

Yes	35.9*	3.0
No	64.1	97.0
Smoking (%):		
Yes	7.7*	24.2
No	92.3	75.8
Alcohol consumption (%):		
Yes	64.1*	87.9
No	35.9	12.1
Chronic conditions (%):		
Cardiovascular disease	87.2*	24.2
Bronchitis, COPD	5.1	9.1
Stomach	25.6	21.2
Diabetes	18.0*	0
Obesity	20.5*	0
Liver	5.1	9.1
Urogenital	20.5*	6.1
Blood	12.8*	3.0
Histological subtype of CRC (%):		
Adenocarcinoma	92.3	-
Others	7.7	
TNM# (%):		
0-I	30.8	
II	38.5	-
III	23.0	
IV	7.7	

Abbreviations: COPD, Chronic obstructive pulmonary disease; TNM, Tumor, node, metastasis. Mann–Whitney U test was used for comparisons, * Significantly different vs. controls.

Table S4. Mean percentages of bacterial genera in the sputum of CRC patients and healthy donors. Mann-Whitney U test.

Genus	CRC	Control	p
Streptococcus	14.22	19.51	> 0.05
Prevotella	8.01	7.66	> 0.05
Nocardia	9.67	7.69	0.005
Dickeya	7.18	7.18	> 0.05
Fusobacterium	3.45	4.21	> 0.05
Leptotrichia	1.92	1.24	0.006
Neisseria	1.23	1.77	> 0.05
Campylobacter	1.79	1.28	0.01
Helicobacter	1.15	0.64	0.0001
Rothia	1.05	0.53	0.003
Treponema	0.91	1.54	0.003
Olsenella	0.84	0.7	> 0.05
Oribacterium	0.59	0.67	0.0002
Porphyromonas	0.56	1.41	0.03
Lachnoanaerobaculum	0.48	0.22	0.01
Tepidibacter	0.44	0.33	> 0.05
Clostridia_UCG-014	0.44	0.22	> 0.05
Clostridium	0.41	0.43	> 0.05
Pseudomonas	0.43	1.36	0.01
Alloprevotella	0.37	0.39	> 0.05
Stomatobaculum	0.34	0.45	> 0.05
Streptobacillus	0.31	1.23	> 0.05
Catonella	0.27	0.26	> 0.05
Parvimonas	0.26	0.36	> 0.05
Saccharimonadales	0.08	0.23	0.005
[Eubacterium]_nodatum_group	0.21	0.09	0.005
Mycoplasma	0.15	0.16	> 0.05
Fretibacterium	0.12	0.19	> 0.05
Abiotrophia	0.09	0.09	> 0.05

Sebaldella	0.06	0.04	> 0.05
Butyrivibrio	0.06	0.33	> 0.05
Veillonella	0.06	0.09	> 0.05
Filifactor	0.05	0.16	> 0.05
Comamonas	0.05	0.02	> 0.05
Defluviitaleaceae_UCG-011	0.05	0.06	> 0.05
[Eubacterium]_brachy_group	0.04	0.06	> 0.05
Bacteroides	0.04	0.03	0.03
Lactobacillus	0.03	0.02	> 0.05
Peptococcus	0.03	0.03	> 0.05
Barnesiella	0.03	0.0003	> 0.05
Mogibacterium	0.03	0.02	> 0.05
Pediococcus	0.02	0.01	> 0.05
Odoribacter	0.01	0.03	> 0.05
Cryptobacterium	0.01	0.01	> 0.05
Shuttleworthia	0.01	0.03	0.03
Atopobium	0.01	0.008	> 0.05
Capnocytophaga	0.01	0.01	> 0.05
Actinomyces	0.01	0.01	> 0.05
Cardiobacterium	0.01	0.01	> 0.05
Staphylococcus	0.01	0.02	> 0.05
Slackia	0.003	0.01	> 0.05
Johnsonella	0.003	0.04	> 0.05
Subdoligranulum	0.002	0.001	> 0.05
Howardella	0.002	0.02	> 0.05
Selenomonas	0.002	0.004	> 0.05
Corynebacterium	0.001	0.0003	> 0.05
Haemophilus	0.001	0.003	> 0.05
Peptostreptococcus	0.0007	0.001	> 0.05
Granulicatella	0.0004	0	> 0.05
Bifidobacterium	0.0003	0.003	> 0.05
Bacillus	0.0002	0	> 0.05
Sneathia	0	0.16	> 0.05
Gemella	0	0.0002	> 0.05
Ruminococcus	0	0.1	> 0.05

↑ - increase compared vs control group; ↓ - decrease compared vs control group.

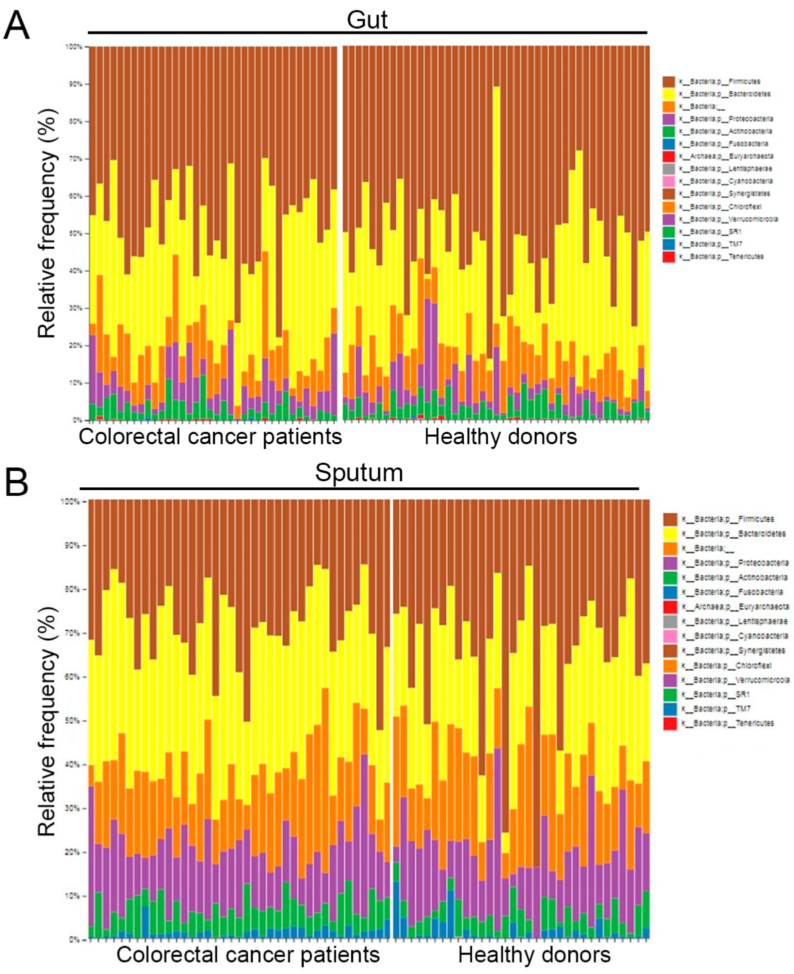

Figure S1. Taxonomic phylum-level structure of gut (A) and sputum (B) microbiomes from CRC patients and healthy donors.

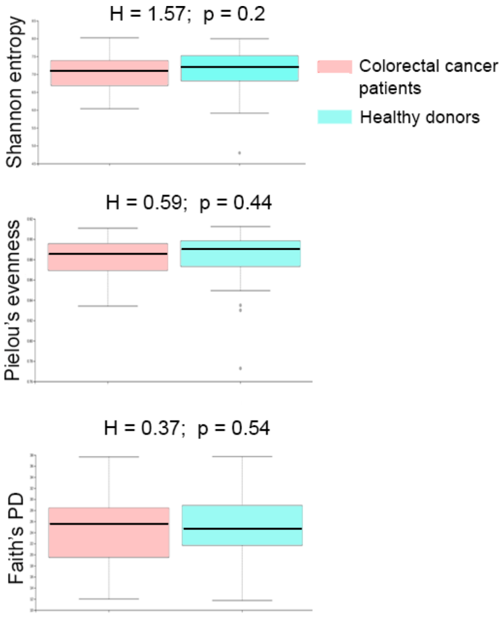

Figure S2. Alpha diversity of gut microbiota from CRC patients and healthy donors (top to bottom: Shannon diversity index, Pielou's index, Faith_pd index).

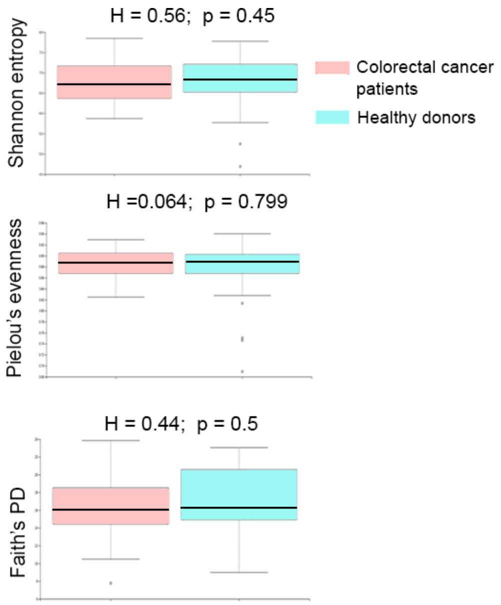

Figure S3. Alpha diversity of sputum microbiota from CRC patients and healthy donors (top to bottom: Shannon diversity index, Pielou's, Faith_pd index).
